# A man-made divide: Investigating the effect of urban–rural household registration and subjective social status on mental health mediated by loneliness among a large sample of university students in China

**DOI:** 10.3389/fpsyg.2022.1012393

**Published:** 2022-11-17

**Authors:** Hui Yu, Shicun Xu, Hui Li, Xiaofeng Wang, Qian Sun, Yuanyuan Wang

**Affiliations:** ^1^Key Laboratory of Brain, Cognition and Education Sciences, Ministry of Education, South China Normal University, Guangzhou, China; ^2^Guangdong Key Laboratory of Mental Health and Cognitive Science, School of Psychology, Center for Studies of Psychological Application, South China Normal University, Guangzhou, China; ^3^Division of Psychology, Faculty of Health and Life Sciences, De Montfort University, Leicester, United Kingdom; ^4^Northeast Asian Research Center, Jilin University, Changchun, China; ^5^Department of Population, Resources and Environment, Northeast Asian Studies College, Jilin University, Changchun, China; ^6^China Center for Aging Studies and Social-Economic Development, Jilin University, Changchun, China; ^7^School of Public Health, Jilin University, Changchun, China

**Keywords:** rural–urban linkages, HuKou system, subjective social status, loneliness, anxiety, depression, college and university students

## Abstract

**Introduction:**

The urban–rural household registration system in China has been documented with profound social consequences in almost all areas of people’s life. This study aims to investigate the underlying mechanism of the rural and urban discrepancies on mental health conditions among a large sample of college students in China.

**Methods:**

A survey was distributed among college students in China. A total of 96,218 college students from 63 colleges completed the survey, answering questions on their urban–rural household registration, disposable household income, subjective social status, feelings of loneliness, and anxiety and depression symptoms. Confirmatory Factor Analysis (CFA) and Structural Equation Modelling (SEM) analyses were conducted, testing the effect of urban–rural registration on one’s mental health, mediated by subjective social status, and loneliness.

**Results:**

Structural Equation Modelling (SEM) results revealed that the urban–rural household registration showed a direct effect on anxiety (*B* = −0.03, 95% CI [−0.038, −0.022], *β* = −0.03, *p* < 0.001) and depression (*B* = −0.03, 95% CI [−0.035, −0.023], *β* = −0.03, *p* < 0.001), indicating that rural household registration had a negative association with anxiety and depression symptoms, albeit the standardised estimate being very small. The indirect path from the urban–rural registration mediated through subjective social status and loneliness to anxiety and depression was both significant, with *B* = 0.01, 95% CI [0.010, 0.010], *β* = 0.01, *p* < 0.001, and *B* = 0.01, 95% CI [0.0090, 0.0090], *β* = 0.01, *p* < 0.001, respectively. The results of the indirect paths demonstrated that students of the rural household registration reported higher anxiety and depression symptoms through a lower subjective social status and higher level of loneliness.

**Conclusion:**

This study indicated that decreasing the disparity of social status and tackling loneliness is the key to improve the overall mental health of college students. The urban–rural household registration system may have a very small direct effect on the college students’ mental health; but students of urban registration enjoyed higher subjective social status, which had a clear protective effect against anxiety and depression symptoms.

## Introduction

The discrepancies between life in urban and rural residents such as household income, access to healthcare infrastructure, physical and mental health, and other important areas in life are documented in many countries including the rich and industrialised ones. For example, according to the 2015 American Community Survey, it has been documented that the median household income for rural households was about 4% lower than the median for urban households in the United States (Bishaw and Posey, 2016). Another study based its analysis on the 2016 March Current Population Survey Public Use Microdata also confirmed that in 2015, 16.7% of the rural population was poor, in comparison to 13.0% of the urban population in the United States (Thiede et al., 2017). On the other hand, some other data suggested the rural as an idyllic place to live, with residents enjoying the beautiful landscapes and more neighbourhood communities, living a happier and healthier life than people living in the urban areas. For example, House et al. (2000) reported that city residents had a mortality hazard rate ratio of 1.62 relative to rural or small town residents, after adjusting for sociodemographic and health variables. It is clear that the urban–rural division is not straightforward, and the regional inequality must be accounted for by multifactorial reasons (McCann, 2020).

The psychological factor closely linked to reginal inequality, or the affluence level of the neighbourhood, is one’s perceived social status. The association between low subjective social status with an array of health problems have been documented, such as physical health (O'Leary et al., 2021), anxiety and depression (Singh-Manoux et al., 2003; Zvolensky et al., 2017). Adler (2009) explicitly claimed that health disparities between Socioeconomic Status (SES) categories could not be explained by poverty in addition to inadequate or lack of health care, but SES went under the skin and had a profound impact on one’s health – the question is how. Indeed, the exact mechanism linking one’s social status and health has not been clearly understood.

One possible pathway is through the feeling of loneliness, being left out and isolated from others (Hughes et al., 2004). Loneliness has been identified as one major risk factor for anxiety and depression (Cacioppo et al., 2006; Ebesutani et al., 2015), and many other health-risky behaviours including suicide attempts (Solmi et al., 2020). On the other hand, loneliness feeling is closely associated with social status: people from lower SES experienced disproportionally high level of loneliness (Domenech-Abella et al., 2017; Macdonald et al., 2018). In addition, subjective social status has shown to be associated with loneliness in the same direction and with similar effect as objectively measured SES (Rubin et al., 2016; Ayalon, 2019). Therefore, it demonstrates that one’s perceived social status is relatively accurate and can be used as a good indicator of one’s actual SES (Adler, 2009). Moreover, loneliness may be an important mediator linking social status and mental health outcomes.

In China, an arbitrary urban–rural household registration system, i.e., *HuKou* system, has been established since 1955, which gives the urban registrants entitlement in better education, housing, jobs, and health care than rural registrants (Wu and Treiman, 2004). The gaps between life of urban and rural registrants have been consistently documented, with urban registrants having higher education attainment and earning more money (China Statistical Yearbook, 2019), reporting better health (Chen et al., 2017), using more health care services (Li et al., 2018), and more. In particular, success in obtaining degrees in the Higher Education sector are crutial for an individuals’ life chances and social position (Hung, 2022). However, due to various cultural and socioenvironmental reasons, treatment and care for people with mental health need have been long under-served (Xu et al., 2018). Only in 2009, severe mental illnesses were incorporated into the national public health service programme (Xu et al., 2014). As a result, not many research has studied the discrepancies of the mental health status between people with urban and rural household registration and their underlying mechanism. The few available studies mainly documented that the prevalence of depression was higher among elderlies of rural than urban registrants (Zhang et al., 2012). However, not much information is available on the urban–rural mental health status of young adults studying at universities.

The present study is the first that aims to examine the effect of urban–rural registration and subjective social status on anxiety and depression symptoms, mediated through feeling of loneliness among university students in China. It is hypothesised that: (1) the urban–rural household registration would have both direct effect and indirect effects through subjective social status and loneliness, on anxiety and depression; and (2) subjective social status would have both direct effect and indirect effect through loneliness, on anxiety and depression.

## Materials and methods

### Participants

Data of this study was part of a cross-sectional survey collected from university students in Jilin Province, China. All students from all the 63 colleges in Jilin Province were invited to the survey; and they were sent the information of the study and a Quick Response (QR) code. Students could quickly go to the survey link from the QR code if they decided to participate. In total, 96,218 students completed and returned the survey between October to November 2021, of which 40,065 were males (41.64%) and 56,153 females (58.36%). In addition, 48,932 (50.86%) of the students were of urban household registration, while 47,286 (49.14%) students were of rural household registration. The mean age of the sample was 19.59 (*SD* = 1.74). The other demographic characters of the sample were summarised in Table 1.

**Table 1:** Summary of demographic characteristics of the sample.

Measured Variable			*n*	%
**Gender**				
	Male		40,065	41.64
	Female		56,153	58.36
**Age**				
	< 18		3,698	3.84
	18–22		86,731	90.14
	23–30		5,640	5.86
	>30		95	0.10
	Missing		54	0.06
**Urban–Rural Registration**				
	Urban		48,932	50.86
	Rural		47,286	49.14
**Religion**				
	None		93,746	97.43
	Buddhism		1,234	1.28
	Christianity		625	0.65
	Islam		232	0.24
	Catholicism		65	0.07
	Taoism		229	0.24
	Others		87	0.09
**Anxiety**				
	GAD total score 0–9		89,741	93.27
	GAD total score > =10		6,477	6.73
		Urban	3,452	7.24
		Rural	3,935	6.21
**Depression**				
	PHQ total score 0–14		92,561	96.20
	PHQ total score > =15		3,657	3.80
		Urban	1,976	4.04
		Rural	1,681	3.55

GAD-7, Generalised Anxiety Disorder Screener; PHQ-9, Patient Health Questionnaire.

All subjects gave their informed consent for inclusion before they participated in the study. The study was conducted in accordance with the Declaration of Helsinki, and the protocol was approved by the Ethics Committee of Jilin University (Project identification code: 2021-9-29).

### Measures

#### Predicting variable

##### Urban–rural household registration

Participants were asked to indicate their household registration being either urban or rural with one question.

##### Subjective social status

Participants were presented with a picture of a 10-rung ladder, and asked to rate where they stand in the Chinese society, with higher rungs indicating higher subjective social status (Singh-Manoux et al., 2003).

#### Mediating variables

##### Loneliness

The Short Scale for Measuring Loneliness (Hughes et al., 2004) was used to measure the participants reported level of loneliness. There were 3 items and they measure how one feels they lack companionship, are left out or isolated from others. The rating was on a 1–3 scale, with 1 = hardly ever, 2 = some of the time, and 3 = often. The total score of loneliness was calculated, ranging from 3 to 9, with a higher score indicating a higher level of perceived loneliness. The internal consistency of the scale was good, with Cronbach’s alpha being 0.82.

#### Outcome variables

##### Anxiety symptoms

The Generalised Anxiety Disorder Screener (GAD-7) (Löwe et al., 2008) was used to measure the participants’ anxiety symptoms. There were 7 items; participants were asked to rate on each symptom on a 0–3 scale based on their experiences during the past 2 weeks, where 0 = not at all, 1 = several days, 2 = more than half of the days, and 3 = nearly every day. The total score of GAD-7 was calculated, ranging from 0 to 21, with a higher score indicating more severe anxiety symptoms. The Cronbach’s alpha was 0.92 in this sample. In Table 1, a cut-off score of > = 10 was used to indicate self-reported moderate or severe level of anxiety symptoms, based on the validation of GAD-7 in a general population (Löwe et al., 2008).

##### Depression symptoms

The Patient Health Questionnaire (PHQ-9) (Kroenke et al., 2001) was used to measure the participants’ depression symptoms. There were 9 items, and similar to the GAD-7, participants were asked to rate on a 0–3 scale based on their experiences during the past 2 weeks. The total score of PHQ-9 was calculated, ranging from 0 to 27, with a higher score indicating more severe depression symptoms. The Cronbach’s alpha was 0.89 in this sample. In Table 1, a cut-off score of > = 15 was used to indicate self-reported moderately severer to severe level of depression symptoms, based on the validation of PHQ-9 in a general population (Kroenke et al., 2001).

#### Covariate

##### Income categories

Participants were asked to indicate the disposable income per family member per year on a 1–6 scale, where 1 = less than 6,000 RMB, 2 = 6,000–14,000 RMB, 3 = 14,000–23,000 RMB, 4 = 23,000-36,000, 5 = 36,000–70,000 RMB, and 6 = more than 70,000 RMB. This scale was adopted from China Statistical Yearbook (2019).

### Data analysis

All analyses were carried out using R version 4.0.3 for Mac. The Confirmatory Factor Analysis (CFA) and the Structural Equation Modelling (SEM) analysis were conducted using the Lavaan package for R (Rosseel, 2012). The dataset was randomly divided into a development and holdout sample (also known as a training and testing sample), with 20% of the original dataset (*N* = 19,244) used for the CFA measurement model fitting, while the other 80% of the original dataset (*N* = 76,974) used for the SEM model testing. This procedure is to reduce the risk of over-fitting the model using the same dataset. Model fit indexes including the Comparative Fit Index (CFI), the Tucker-Lewis Index (TLI), and the Root Mean Square Error of Approximation (RMSEA) were used to assess the goodness of the model fit. It is commonly considered that a CFI/TLI higher than 0.95, and a RMSEA smaller than 0.05, indicate a good model fit. As the sample size is so large, only *p* < 0.001 was considered statistically significant in this study.

## Results

### Descriptive statistics

The sample is consisting of 96,218 university students, among which 50.86% are from urban household and 49.14% from rural household. The means and SDs of the study variables by urban–rural registration were summarised in Table 2. In addition, Welch two sample t-tests were conducted to test whether there was a significant difference between the urban and rural groups on the study variables. Results showed that students of urban household registration reported a significantly higher disposable income than those of rural registration, *t*(93243) = 81.60, *p* < 0.001, and the effect size was moderate, with Cohen’s d = 0.50. Students of urban household registration also reported a moderate effect sized higher subjective social status than students of rural registration, *t*(96130) = 71.45, *p* < 0.001, Cohen’s d = 0.45. The *t*-test result also showed that students of urban registration reported lower loneliness than students of rural registration, *t*(9611) = −4.15, *p* < 0.001; however, the effect size was negligible, with Cohen’s *d* = 0.02. The students of urban and rural household registration reported similar level of anxiety and depression symptoms, measured by GAD-7 and PHQ-9 total scores, respectively. Although it must be pointed out that Chi-squre tests revealed a significantly higher percentage among urban students than rural students scoring on the high end of the anxiety (GAD > = 10) and depression (PHQ > = 15) scales (see Table 1), *χ*^2^ (*df* = 1) = 40.77, *p* < 0.001, and *χ*^2^ (*df* = 1) = 15.36, *p* < 0.001, for anxiety and depression symptoms, respectively.

**Table 2:** Means and SDs of the study variables by urban–rural registration.

	Total *N* = 96,218		Urban *N* = 48,932		Rural *N* = 47,286		Welch Two-Sample t-test	Cohen’s d
	Mean	SD	Mean	SD	Mean	SD	IV: urban–rural group	
Income Category [range 1–6]	2.45	1.41	2.80	1.50	2.09	1.21	***t*(93243) = 81.60, ***p*** < 0.001**	**0.50**
Subjective Social Status [range 1–10]	4.47	1.63	4.83	1.60	4.10	1.59	***t*(96130) = 71.45, *p* < 0.001**	**0.45**
Loneliness [range 3–9]	4.59	1.57	4.57	1.57	4.61	1.57	***t*(96122) = −4.15, *p* < 0.001**	**0.02**
Anxiety [range 0–21]	3.69	3.89	3.72	3.97	3.66	3.80	*t*(96204) = 2.27, *p* = 0.023	0.02
Depression [range 0–27]	5.13	4.47	5.13	4.54	5.13	4.39	*t*(96216) = −0.16, *p* = 0.870	0.00

The reference group of the Welch *t*-test was the rural household registration. *p* < 0.001 indicates a statistically significant result. Significant *t*-test results were highlighted bold.

### CFA measurement model

**Figure 1:**
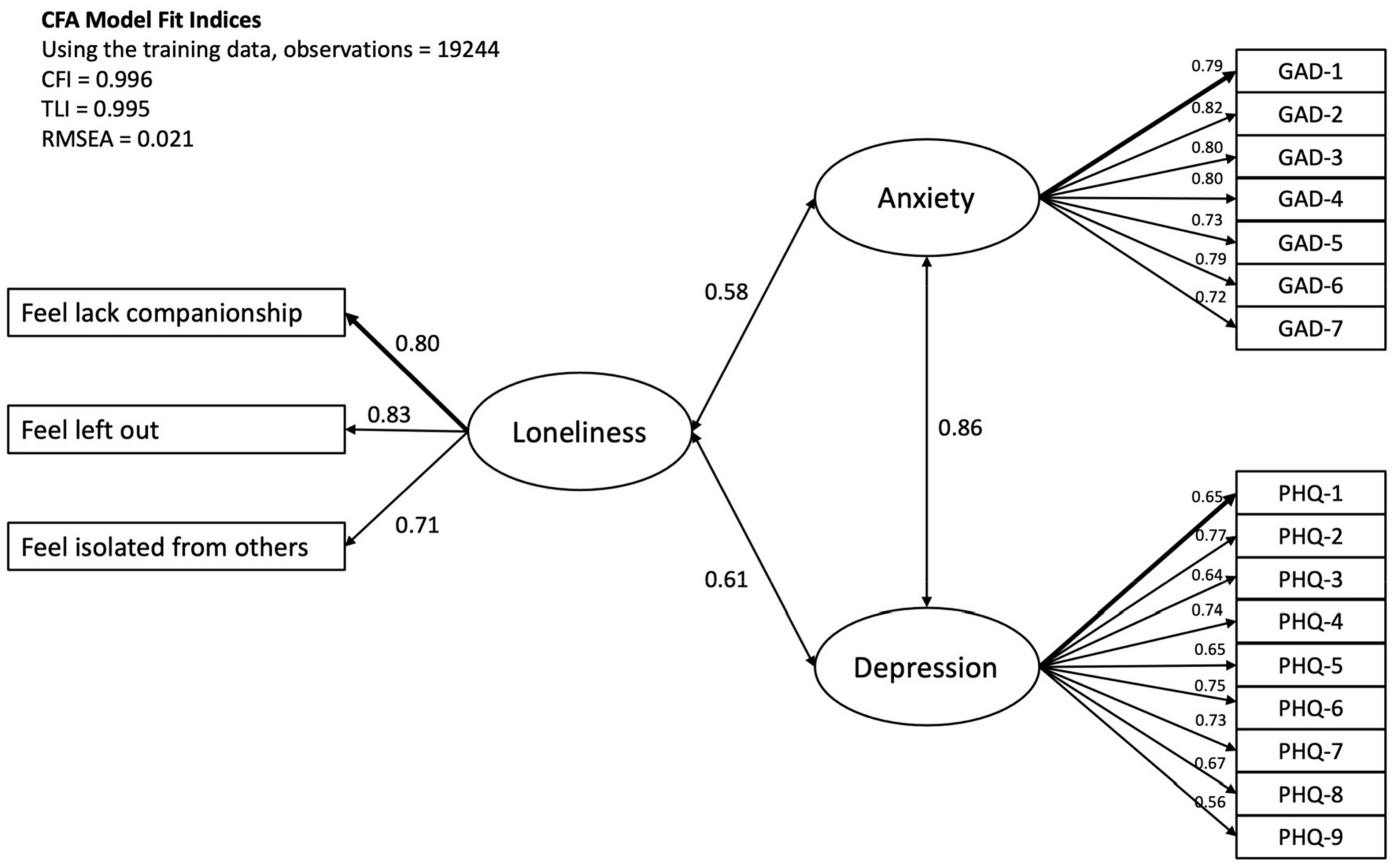
The CFA measurement model of loneliness, anxiety, and depression. The standardised loading estimates were shown in the figure, and all paths were significant with *p* < 0.001.

A CFA model of loneliness, anxiety, and depression measured by the Short Scale for Measuring Loneliness, GAD-7, and PHQ-9 was first established by the training sample, consisting of 20% of the original dataset. The model fit indices indicated an adequate fit, with CFI = 0.996, TLI = 0.995, and RMSEA = 0.021. The standardised factor loading estimates ranged from 0.56 to 0.83, and all the measured items significantly loaded to their latent construct, with *p* < 0.001. The details of the path estimates were summarised in Table 3 and Figure 1.

**Table 3:** CFA measurement model of loneliness, anxiety, and depression.

	Factor indication	Estimate	SD	Standardised estimate	*p*-value
**Loneliness**					
Item-1	Feel lack companionship	1.00		0.80	
Item-2	Feel left out	1.03	0.01	0.83	< 0.001
Item-3	Feel isolated from others	0.76	0.01	0.71	< 0.001
**Anxiety**					
GAD-1	Feeling nervous, anxious or on edge	1.00		0.79	
GAD-2	Not being able to stop or control worrying	1.10	0.01	0.82	< 0.001
GAD-3	Worrying too much about different things	1.12	0.01	0.80	< 0.001
GAD-4	Trouble relaxing	1.10	0.01	0.80	< 0.001
GAD-5	Being restless and hard to sit still	0.80	0.01	0.73	< 0.001
GAD-6	Being easily annoyed or irritable	1.01	0.01	0.79	< 0.001
GAD-7	Feeling afraid as if something awful might happen	0.81	0.01	0.72	< 0.001
**Depression**					
PHQ-1	Little interest or pleasure in doing things	1.00		0.65	
PHQ-2	Feeling down, depressed, or hopeless	1.14	0.01	0.77	< 0.001
PHQ-3	Trouble falling or staying asleep, or sleeping too much	1.14	0.02	0.64	< 0.001
PHQ-4	Feeling tired or having little energy	1.21	0.02	0.74	< 0.001
PHQ-5	Poor appetite or overeating	1.10	0.02	0.65	< 0.001
PHQ-6	Feeling bad about oneself	1.21	0.02	0.75	< 0.001
PHQ-7	Trouble concentrating on things	1.25	0.02	0.73	< 0.001
PHQ-8	Moving or speaking very slowly or being very fidgety	0.92	0.02	0.67	< 0.001
PHQ-9	Thoughts that you would be better off dead or of hurting yourself	0.57	0.01	0.56	< 0.001

*p* < 0.001 indicates a statistically significant loading.

### SEM testing model

An SEM model testing the urban–rural registration predicting anxiety and depression, mediated by perceived social status and loneliness and with sex and income as covariates was conducted by the testing sample, consisting of 80% of the original dataset. The model fit indices indicated an adequate fit, with CFI = 0.995, TLI = 0.995, and RMSEA = 0.020. The analysis revealed that the urban–rural household registration showed a direct effect on anxiety (B = -0.03, 95% CI [−0.038, −0.022], β = −0.03, *p* < 0.001) and depression (B = −0.03, 95% CI [−0.035, −0.023], β = −0.03, *p* < 0.001), indicating that rural household registration was negatively associated with anxiety and depression symptoms, albeit the standardised estimate being very small. On the other hand, students of rural household registration reported a significantly lower subjective social status than those of urban registration (*B* = −0.41, 95% CI [−0.42, −0.38], *β* = −0.12, *p* < 0.001), while subjective social status had a protective effect against anxiety (*B* = −0.02, 95% CI [−0.023, −0.019], *β* = −0.06, *p* < 0.001) and depression (*B* = −0.03, 95% CI [−0.030, −0.026], *β* = −0.10, *p* < 0.001). Subjective social status had a negative effect on loneliness (*B* = −0.04, 95% CI [−0.043, −0.039], *β* = −0.13, *p* < 0.001), meaning the higher the subjective social status, the lower level of reported loneliness; while loneliness was positively associated with anxiety (*B* = 0.61, 95% CI [0.60, 0.62], *β* = 0.58, *p* < 0.001) and depression (*B* = 0.53, 95% CI [0.52, 0.54], *β* = 0.61, *p* < 0.001).

Sex and income were used as covariates in the model. Sex was significantly associated with all predicting, mediating and outcome variables, with females reporting a higher subjective social status (*B* = 0.27, 95% CI [0.24, 0.29], *β* = 0.08, *p* < 0.001), greater loneliness (*B* = 0.04, 95% CI [0.031, 0.047], *β* = 0.04, *p* < 0.001), greater anxiety (*B* = 0.05, 95% CI [0.045, 0.061], *β* = 0.05, *p* < 0.001) and depression symptoms (*B* = 0.03, 95% CI [0.019, 0.031], *β* = 0.03, *p* < 0.001) than males. Income was also significantly associated with higher subjective social status (*B* = 0.45, 95% CI [0.44, 0.45], *β* = 0.38, *p* < 0.001), greater anxiety (*B* = 0.01, 95% CI [0.010, 0.014], *β* = 0.03, *p* < 0.001), and depression (*B* = 0.01, 95% CI [0.0060, 0.010], *β* = 0.03, *p* < 0.001) symptoms.

The indirect paths from the urban–rural registration mediated through subjective social status and loneliness to anxiety and depression were both significant, with *B* = 0.01, 95% CI [0.010, 0.010], *β* = 0.01, *p* < 0.001, and *B* = 0.01, 95% CI [0.0090, 0.0090], *β* = 0.01, *p* < 0.001, respectively. The results of the indirect paths demonstrated that students of the rural household registration reported higher anxiety and depression symptoms through a lower subjective social status and higher level of loneliness. The total effect of urban–rural household registration on anxiety and depression (*B* = −0.02, 95% CI [−0.028, −0.012], *β* = −0.02, *p* < 0.001, and *B* = −0.02, 95% CI [−0.029, −0.013], *β* = −0.02, *p* < 0.001 respectively), however, indicated that rural registration was negatively associated with mental health conditions.

**Figure 2:**
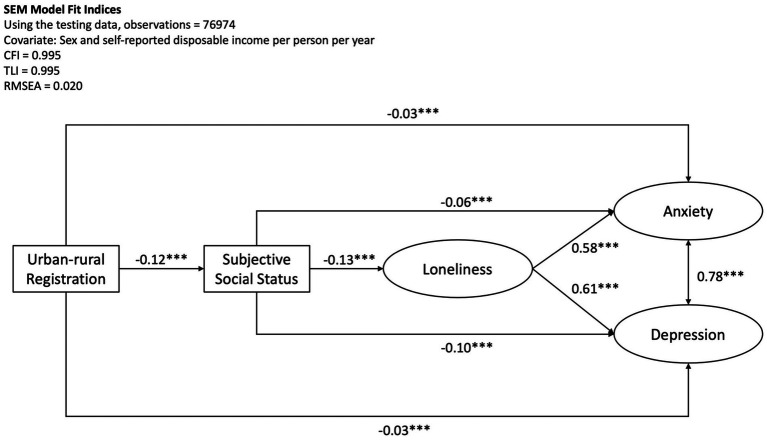
SEM model testing urban–rural registration predicting anxiety and depression mediated by subjective social status and loneliness. The standardised path estimates were shown in the figure, and all paths were significant with p < 0.001. Sex and self-reported disposable income were covariates in the model.

The indirect paths from the subjective social status to anxiety and depression through loneliness were also significant, with *B* = −0.02, 95% CI [−0.027, −0.023], *β* = −0.07, *p* < 0.001, and *B* = −0.02, 95% CI [−0.024, −0.020], *β* = −0.08, *p* < 0.001, respectively. The indirect paths demonstrated that students with higher subjective social status reported lower anxiety and depression through feeling lower level of loneliness. The total effect of subjective social status on anxiety and depression (*B* = −0.04, 95% CI [−0.046, −0.042], *β* = −0.13, *p* < 0.001, and *B* = −0.05, 95% CI [−0.050, −0.046], *β* = −0.18, *p* < 0.001 respectively) also indicated that subjective social status had an overall protective effect against mental health conditions. The details of the path estimates were summarised in Table 4 and Figure 2.

**Table 4:** SEM testing model: Urban–rural registration predicting anxiety and depression, mediated by subjective social status and loneliness.

Path		Estimate	SD	95% CI	Standardised estimate	*p*-value
**Regression path**						
**Urban–rural registration →**						
	Subjective Social Status	−0.40	0.01	[−0.42, −0.38]	−0.12	< 0.001
	Anxiety	−0.03	0.00	[−0.038, −0.022]	−0.03	< 0.001
	Depression	−0.03	0.00	[−0.035, −0.023]	−0.03	< 0.001
**Subjective Social Status →**						
	Loneliness	−0.04	0.00	[−0.043, −0.039]	−0.13	< 0.001
	Anxiety	−0.02	0.00	[−0.023, −0.019]	−0.06	< 0.001
	Depression	−0.03	0.00	[−0.030, −0.026]	−0.10	< 0.001
**Loneliness →**						
	Anxiety	0.61	0.01	[0.60, 0.62]	0.58	< 0.001
	Depression	0.53	0.01	[0.52, 0.54]	0.61	< 0.001
**Correlation path**						
	Anxiety ↔ Depression	0.12	0.00	[0.12, 0.12]	0.78	< 0.001
**Covariate path**						
	Income → Subjective Social Status	0.45	0.00	[0.44, 0.45]	0.39	< 0.001
	Income → Perceived Loneliness	0.00	0.00	[−0.0059, −0.0019]	0.00	0.220
	Income → Anxiety	0.01	0.00	[0.010, 0.014]	0.03	< 0.001
	Income → Depression	0.01	0.00	[0.0060, 0.010]	0.03	< 0.001
	Sex → Subjective Social Status	0.27	0.01	[0.24, 0.29]	0.08	< 0.001
	Sex → Perceived Loneliness	0.04	0.00	[0.031, 0.047]	0.04	< 0.001
	Sex → Anxiety	0.05	0.00	[0.045, 0.061]	0.05	< 0.001
	Sex → Depression	0.03	0.00	[0.019, 0.031]	0.03	< 0.001
**Indirect path**						
**U/R** → **SSS** → **Loneliness** →						
	Anxiety	0.01	0.00	[0.010, 0.010]	0.01	< 0.001
	Depression	0.01	0.00	[0.0090, 0.0090]	0.01	< 0.001
**SSS** → **Loneliness** →						
	Anxiety	−0.02	0.00	[−0.027, −0.023]	−0.07	< 0.001
	Depression	−0.02	0.00	[−0.024, −0.020]	−0.08	< 0.001
**Total effect**						
	U/R → Anxiety	−0.02	0.00	[−0.028, −0.012]	−0.02	< 0.001
	U/R → Depression	−0.02	0.00	[−0.029, −0.013]	−0.02	< 0.001
	SSS → Anxiety	−0.04	0.00	[−0.048, −0.044]	−0.13	< 0.001
	SSS → Depression	−0.05	0.00	[−0.051, −0.047]	−0.18	< 0.001

U/R, urban–rural household registration; SSS, subjective social status.

The urban group was the reference group in the analysis. Income and sex were used as covariates in the model.

*p* < 0.001 indicates a statistically significant path.

## Discussion

The results of SEM analysis supported both hypotheses: (1) the urban–rural household registration had a significant direct effect and indirect effect through subjective social status and loneliness on anxiety and depression symptoms; and (2) subjective social status had a significant direct effect and indirect effect through loneliness on anxiety and depression symptoms.

It was curious that the rural registration showed a positive association to anxiety and depression through lower subjective social status and higher self-reported loneliness path; and the rural registration also showed a negative association to anxiety and depression through the direct path, which resulted in an overall negative association between the rural registration and mental health conditions. This was surprising and in contrast to the commonly perceived image of the urban–rural discrepancies in China, which almost always indicated that people of urban registration would be better-off than those of rural registration in all documented areas (Wu and Treiman, 2004; Zhang et al., 2012; Xu et al., 2018). *HuKou* system posts strict restrictions of people’s access to education, health care, insurance, and even purchasing houses in an area that is other than the registered location (Xu et al., 2014). As the migration route within China is almost exclusively from rural to cities, this restriction mainly disfavours people with rural household registration. Therefore, unlike in the Western countries, where within-country movement is free, the Chinese urban–rural disparities were mostly viewed as unequivocally advantageous to urban registrants.

However, the result of this study seemed to be contradicting this common understanding by showing a positive effect of rural registration against mental health conditions. The result, however, must be put into perspective. Firstly, the simple two-sample t-test indicated that there was no significant difference between students of urban and registration on reported anxiety and depression symptoms. Secondly, when the relevant psychological factors were put into the model, the urban–rural registration showed significant effect on anxiety and depression symptoms. However, the standardised path coefficients for the direct effect and total effect of urban–rural registration on anxiety and depression were very small in scale. Lastly, the sample of this study mainly comprised of university students who were full of hopes and did not have much life experience yet. Therefore, those young adults of rural registration might not have had encountered real life challenges due to *HuKou* restriction yet, such as finding a job, buying a house etc. Treiman (2012) showed that the group of people of rural registration living in the cities occupied less nonmanual jobs, earned less money, possessed fewer amenities in their household than people of urban registration. It has also been documented that people with rural *HuKou* but lived in the cities reported higher depression symptoms than those who had urban *HuKou* (Song and Smith, 2019). It might be a matter of time for the gap in the “predicted direction” to appear when people of rural registration reported higher level of stress.

On the other hand, the positive effect of green spaces on one’s mental health has been firmly confirmed by research and incorporated into urban design (Brown et al., 2018). Our result might be useful to indicate that majority of students of rural registration still benefit from the closeness to nature at a young age. In particular, our data documented that urban students were more likely than rural students to score on the high end of the anxiety and depression scales. There was also evidence to show that elderly rural residents had high level of community support and reported relatively good health (Xu et al., 2021). In addition, there has been increasing evidence that the gap between life in rural and urban areas has been decreasing (Treiman, 2012; Zhang and Treiman, 2013). The current policy changes also helped to change people’s attitude to the value of rural *HuKou* by giving the rural residence some exclusive benefits (Chen and Fan, 2016). Although the institutional barrier of the *HuKou* system to restrict people of the rural registration from upward social mobility has been great, there is hope that tertiary education might help the hopeful young adults to pursue a desirable life wherever they choose with freedom.

Regarding the effect of subjective social status on anxiety and depression, the result of this study supported existing literature that higher social status predicted a lower level of loneliness and therefore, was association with lower anxiety and depression symptoms. It has been well documented that loneliness is unequal among the population with different SES background, with lower SES associated to higher perceived loneliness (Qualter et al., 2021). In our sample, in comparison to the better-off peers, the young students from poorer families might feel it more difficult to make firends when there are financial concerns: for example, they may be more reluctant to go out with their peers for social events. Although in the oriental context, the alarmingly high prevalence of loneliness and their underlying mechnism have not been fully understood, it was reported to be associated with and moderated by level of income (Badman et al., 2022). The result of our model clearly indicated a link between low subjective social status and greater feelings of loneliness.

Martín-María et al. (2021) showed that phone call programs tackling loneliness feeling could significantly reduce one’s reported anxiety and depression symptoms. Social prescribing by clinical professionals as a means to combact loneliness in the modern society by linking individuals to the local non-clinical providers in the community has become more common in many Western societies (Liebmann et al., 2022). The result from the current study also showed that loneliness was positively associated with anxiety and depression with significant path coefficients, adding to the ever growing literature that loneliness plays an important role in mental health conditions (Erzen and Çikrikci, 2018; Wilkialis et al., 2021). In addition, higher subjective social status also had a direct protective effect against anxiety and depression. In particular, as students of the rural *HuKou* reported a significantly lower subjective social status, the indirect effect from the urban–rural registration through subjective social status and loneliness proved disadvantageous to those with rural *HuKou*. Song and Smith (2019) showed that people with rural registration endured the highest level of hardships and adversities throughout life; those who had converted their rural registration to urban registration and lived in cities had better income and fewer hardships than those who continued living in the rural areas, but were disadvantaged in a variety of health measures than those who were born with urban registration. Importantly, the cited study showed that these differences could be mainly accounted for by differences in socioeconomic status. The result of our study added new evidence to linking the urban–rural division as well as subjective social status to mental health outcomes.

In the model, sex and income were uses as covariates. Results indicated that both covariates were important in the model with most path coefficients being significant and in the predicted directions. In particular, females reported significantly higher subjective social status, greater feeling of loneliness, greater anxiety and depression symptoms. These results were in line with a great amount of literature documenting the gender difference of loneliness (Rokach, 2018) and mental health (Hallers-Haalboom et al., 2020; Hyde and Mezulis, 2020), but the reported higher subjective social status may need some explanation. The income gap favouring males over females has been firmly established in all studies areas, and China is no exception. However, there has been a tradition of materially pampering daughters more than sons in some areas of China (Kim et al., 2018), which might have lead to the observed positive association that females reported higher subjective social status than males. Conversely, this may also be the simple result that females inflated their social status more than males in this context (Kennedy and Kray, 2022). The mechanism behind this observation is beyond this study’s scope, but this result would lead to interesting future research examining the gender similarities and differences in the association between real income, subjective social status and mental health conditions.

The major limitation of this study is that it is a cross-sectional study using a group of young adults who are still studying in universities. As discussed, it is of vital importance to understand whether over time, these bunch of hopeful young adults would experience higher level of mental health conditions, and to what extent, the arbitrary urban–rural household registration system would inhibit the rural registrants from getting the life they wish to realise. A longitudinal follow-up study in different stages of their lives would be most powerful to answer the above-mentioned questions. However, this study provides an important puzzle piece that contributes significantly to understand the whole picture of the health disparities in Chinese society.

## Conclusion

In conclusion, this study clearly demonstrated the complexity of the effect of urban–rural household registration on mental health in China. However, it was straightforward that decreasing the disparity of social status is the key to improve the overall mental health of university students. In addition, loneliness is an important pathway linking social status and mental health. Intervention programs tackling loneliness would yield positive results in decreasing anxiety and depression.

## Data availability statement

The raw data supporting the conclusions of this article will be made available by the corresponding authors on reasonable request.

## Ethics statement

The studies involving human participants were reviewed and approved by Jilin University. The patients/participants provided their written informed consent to participate in this study.

## Author contributions

HY, YW, and SX were responsible for the conception, organisation, and execution of the study and manuscript revision. SX, XW, QS, and HL were responsible for the data collection. HY was responsible for the statistical analysis and verification of the underlying data. HY and YW were responsible for the manuscript preparation. All authors had full access to all the data in the study and confirmed their responsibility for the decision to submit it for publication.

## Funding

This study was founded by the National Natural Science Foundation of China (NSFC) Grant number 82201708.

## Conflict of interest

The authors declare that the research was conducted in the absence of any commercial or financial relationships that could be construed as a potential conflict of interest.

## Publisher’s note

All claims expressed in this article are solely those of the authors and do not necessarily represent those of their affiliated organizations, or those of the publisher, the editors and the reviewers. Any product that may be evaluated in this article, or claim that may be made by its manufacturer, is not guaranteed or endorsed by the publisher.
